# Fatigue and sleepiness determine respiratory quality of life among veterans evaluated for sleep apnea

**DOI:** 10.1186/s12955-017-0624-x

**Published:** 2017-03-14

**Authors:** Denis Vinnikov, Paul D. Blanc, Alaena Alilin, Moshe Zutler, Jon-Erik C. Holty

**Affiliations:** 1 Department of Internal Medicine, Occupational Diseases and Hematology, Kyrgyz State Medical Academy, Bishkek, Kyrgyzstan; 2 School of Public Health, Al-Farabi Kazakh National University, Almaty, Kazakhstan; 3 Division of Occupational and Environmental Medicine, Department of Medicine, University of California, San Francisco, San Francisco, CA USA; 4 SF Veterans Affairs Medical Center, San Francisco, CA USA; 5 Pulmonary, Critical Care and Sleep Medicine Section, VA Palo Alto Health Care System, Palo Alto, CA USA; 6 Starling Physicians, New Britain, CT USA; 7 Division of Pulmonary and Critical Care Medicine, Department of Medicine, Stanford University, Palo Alto, CA USA; 8 Center for Health Policy (CHP/PCOR), Stanford University, Palo Alto, CA USA

**Keywords:** Quality of life, Sleep apnea syndromes, Lung diseases, Disorders of excessive somnolence, Fatigue, Health status indicators, Pulmonary disease, Chronic obstructive, Asthma

## Abstract

**Background:**

In those with symptoms indicative of obstructive sleep apnea (OSA), respiratory-specific health-related quality of life (HRQL) may be an important patient-centered outcome. The aim of this study was to assess the associations between sleepiness, fatigue, and impaired general and respiratory-specific HRQL among persons with suspected OSA.

**Methods:**

We evaluated military veterans consecutively referred for suspected OSA with sleep studies yielding apnea-hypopnea index (AHI) values. They also completed the sleepiness (Epworth Sleepiness Scale [ESS]), and fatigue (Fatigue Severity Scale [FSS]) questionnaires, as well as two HRQL instruments (the generic Short-Form SF-12v2 yielding the Physical Component Scale [PCS] and the respiratory-specific Airways Questionnaire [AQ]-20R). Multiple linear regression tested the associations between ESS and FSS (standardized as Z scores for scaling comparability) with AQ-20R, accounting for AHI, SF-12v2-PCS and comorbid respiratory conditions other than OSA.

**Results:**

We studied 1578 veterans (median age 61.1 [IQR 16.8] years; 93.9% males). Of these, 823 (52%) met AHI criteria for moderate to severe OSA (AHI ≥15/h). The majority reported excessive daytime sleepiness (53%; median ESS 11 [IQR 9]) or fatigue (61%; median FSS 42 [IQR 23]). The median AQ-20R was 4 [IQR 1–8]. Controlling for AHI, SF-12v2-PCS, respiratory co-morbid conditions, body mass index, and demographics, both ESS and FSS were significantly associated with poorer AQ-20R: for each; ESS, 1.6 points (95% CI 1.4–1.9), and for FSS, 2.5 points (95% CI, 2.3–2.7).

**Conclusions:**

Greater daytime sleepiness and fatigue are associated with poorer respiratory-specific HRQL, over and above the effects of OSA, respiratory comorbidity, and generic physical HRQL.

## Background

Health-related quality of life (HRQL) is a critical patient-centered outcome measuring generic or disease-specific health status. Disease-specific HRQL is relevant to chronic health conditions whose effects are manifested through discrete subjective symptoms and limitations. Respiratory-specific HRQL is a condition-specific construct emphasizing patient-perceived impacts related to dyspnea and other pulmonary limitations.

Obstructive sleep apnea (OSA) is a common respiratory condition occurring during sleep that carries substantial morbidity and mortality [1]. Although many OSA patients report symptoms such as snoring, general population and sleep clinic-based studies suggest many do not report excessive daytime sleepiness or fatigue [1–4]. Furthermore, the relationship between OSA and general (and particularly physical) [5–11] as well as respiratory-specific HRQL [12] is less well established. On the other hand, respiratory disorders such as chronic obstructive pulmonary disease (COPD), independent of OSA, are associated with poor subjective and objective sleep quality [13, 14], sleepiness [2] and decreased general and respiratory specific HRQL [15]. Furthermore, sleep disturbance is a major determinant of HRQL in those with chronic respiratory disorders such as asthma [16] or COPD [17].

The determinants of respiratory-specific HRQL in OSA (with or without co-morbid lung conditions) remain to be well characterized [12, 18, 19]. Sleepiness and fatigue as HRQL determinants are particularly relevant given they may adversely impact patient-perceived physical and respiratory-specific HRQL status [20–22]. The extent to which OSA, comorbid respiratory disorders, daytime sleepiness and fatigue in combination may contribute to HRQL remains unclear. The aim of this study was to test sleepiness and fatigue as independent predictors of respiratory-specific HRQL among persons with symptoms suggestive of OSA who went on to diagnostic testing. This has been an open question whose answer is relevant clinically to help better gauge the likely impact of OSA diagnosis and treatment on HRQL among symptomatic patients. Our primary hypothesis was that daytime sleepiness and fatigue in this population would be independently associated with respiratory-specific HRQL, taking into account OSA as well as concomitant respiratory disease.

## Methods

This cross-sectional retrospective study of prospectively collected clinical data was undertaken in a cohort of military veterans referred due to OSA symptoms for a standardized assessment protocol that included both a multi-battery questionnaire and confirmatory polysomnography. We intentionally included a respiratory-specific HRQL measure in the questionnaire so that we could clinically assess breathing problems as well as carry out an analysis with this as our central patient-centered outcome.

### Study subjects

Study participants were referred for OSA assessment based on their symptoms according to routine clinical practice and the potential subject pool comprised all veterans referred to the Veterans Affairs (VA) Palo Alto Healthcare System’s Pulmonary-Sleep Section for evaluation of complaints of either disrupted sleep or snoring who completed formal sleep study testing. Thus, participant accrual was passive: there was no specific recruitment or outreach for the study itself. Data from consecutive subjects aged 21–95 studied from May 2011 through July 2014 were retrospectively analyzed. Subjects were eligible for study inclusion if they completed a structured sleep questionnaire and an overnight diagnostic sleep study. Exclusion criteria included: incomplete or missing questionnaire or inadequate sleep study data; evidence of a physiologic sleep disorder other than OSA (e.g., narcolepsy, periodic limb movements, central sleep apnea [central apnea index ≥5/h]); supplemental oxygen provided during diagnostic sleep study; or a sleep questionnaire/study completed during a period of hospitalization. The study protocol was approved (with a waiver of consent) by Stanford University’s institutional review board and the VA Palo Alto Heath Care System’s Research and Development committee with annual reviews. The sleep questionnaire and sleep study were part of the existing medical record, and all patient health information was de-identified and stored on VA approved servers behind existing firewalls and accessible only via user logins of the approved investigators. Veterans received clinical follow-up based on a detailed examination of sleep questionnaire and sleep study results by a staff VA sleep clinician.

### Survey data and instrument scoring

Subjects self-completed a structured hard-copy questionnaire prior to their overnight sleep study examination. The questionnaire elicited: socio-demographics, smoking status, relevant medical history. It also included generic and respiratory-specific HRQL instruments and standard batteries assessing daytime sleepiness and fatigue. We assessed generic HRQL using the Medical Outcomes Survey Short Form-12, version 2 (SF-12v2) instrument, a validated general HRQL measure that has been widely used, including in patients with OSA, asthma, and COPD [23]. The Physical Component Score (PCS) was calculated from the SF-12v2 responses. Respiratory-specific HRQL was measured using the Airways Questionnaire 20 Revised (AQ-20R), a 20-question instrument (possible range 0 to 20) developed to measure respiratory-specific HRQL, with higher scores indicating worse HRQL [24]. The AQ-20R has been used to assess respiratory-specific HRQL across a range of pulmonary conditions, including asthma and COPD [24, 25]. Daytime sleepiness was assessed using the 8-item (possible range 0 to 24) Epworth Sleepiness Scale (ESS); a composite score of >10 was used to define sleepiness [26]. Fatigue was measured using the 9-item (possible range 9 to 63) Fatigue Severity Scale (FSS); a composite score of >36 was used to define the presence of daytime fatigue [27].

### Assessment of overnight polysomnography

Sleep apnea evaluations were performed using two types of studies: Type-III portable sleep apnea testing using an Embletta X100 (Embla, Broomfield, CO) that includes nasal pressure transducer, thoracoabdominal movement detection, pulse oximeter, single-lead electrocardiogram (ECG), actigraphy and body position; and Type-I monitored, in-lab polysomnography (PSG) using Alice-5 software (Respironics, Murraysville, PA), that includes all data channels recorded on the Embletta X100 with the addition of electroencephalogram (EEG), electrooculogram, and electromyogram. Selection of portable verses in-lab PSG followed American Academy of Sleep Medicine (AASM) guidelines for portable OSA diagnosis and depended on availability, pretest OSA probability and consideration of comorbid respiratory, cardiac, and neuromuscular disorders [28]. During the 38-month period, approximately 80% of the studies were portable; the remaining 20% were performed at the sleep laboratory. Patients with an AHI <5/h on a portable sleep study were offered a monitored PSG to confirm the absence of OSA.

Sleep study data from the overnight sleep studies were manually scored and reviewed by a sleep-medicine boarded physician. Sleep study scoring conformed to AASM guidelines [29]. Apneas (cessation of airflow ≥90% of baseline for ≥10 s) and hypopneas (≥30% reduction in baseline airflow for ≥10 s accompanied by a ≥4% reduction in oxyhemoglobin saturation) are combined to calculate an apnea-hypopnea index (AHI; number of apneas + hypopneas per hour). Sleep apnea was defined as being present if the AHI was ≥5 events per hour [1]. Mild sleep apnea was defined as an AHI between 5 and less than 15 events per hour; moderate sleep apnea as an AHI between 15 and less than 30 events per hour; severe sleep apnea as an AHI ≥30 events per hour.

### Medical record review

A self-reported diagnosis of lung disease (asthma, COPD or interstitial lung disease) was subsequently confirmed by electronic medical record (EMR) review by a pulmonologist who considered prior pulmonary function testing, chest radiography, smoking history, and reported clinical respiratory symptoms and documented physical examination findings in the diagnostic validation. Height and weight measurements were obtained from the last inputted data from the EMR to calculate a body mass index (BMI). We defined obesity as a BMI ≥30 kg/m^2^. In addition, EMR review supplemented self-report of smoking status.

### Analysis

Statistical analysis used NCSS software, version 9 (Kaysville, UT). All continuous variables in this analysis, including age, BMI, AHI, ESS, FSS, AQ-20R and SF-12 PCS, had non-normal distributions based on the Shapiro-Wilk test for normality. Consistent with these distributions, summary descriptive data report median values and their corresponding interquartile ranges (IQRs). Differences in descriptive characteristics by OSA presence or absence were tested by chi square, t-test, or Wilcoxon rank sum test as appropriate to the data. We used multivariable linear regression including demographic covariates to estimate the associations between ESS, FSS, and AHI as independent variables and SF-12v2-PCS and AQ20-R as dependent variables. For ESS and FSS, we repeated these analyses stratified by the presence or absence of OSA and, in a separate analysis, stratified by obesity. To ensure uniformity of fatigue and sleepiness scaling, z-scores of these variables were generated (each variable divided by its standard deviation). In order to assess whether the relationship between ESS and FSS and respiratory-specific HRQL was independent of demographics, BMI, OSA severity, comorbid lung disease, and generic HRQL (SF-12 PCS, also scaled in z-scores), we tested a multiple logistic regression model that included all of these covariates as independent covariates. The outcome of poorer respiratory-specific QOL was defined as the highest quintile of observed AQ-20R scores in the cohort (scored 11 through 20). As a sensitivity analysis, we further restricted this analysis to the subset of those with in-laboratory monitored sleep studies.

We worked together as collaborative research team. Our data analysis strategy was driven by our central aim to study the determinants of respiratory-specific HRQL in persons with symptoms leading to an evaluation for sleep apnea. For this reason, we formulated an approach to assess fatigue and sleepiness as key predictors of HRQL, while taking into account the actual determination of OSA by polysomnography. We did not deviate substantively from this strategy. The covariates of interest were tested and presented in the relevant multivariate models.”

## Results

Of the 1578 persons analyzed, 823 (52.1%) met criteria for moderate to severe OSA (AHI ≥15/h), whereas 755 (47.9%) manifested mild (*n* = 451, AHI < 15/h and ≥5/h) or no OSA (*n* = 304, AHI < 5/h) (Table 1). Just under one-third of the study participants met criteria for severe OSA (30.5%). Demographic data by OSA status are presented in Table 1. Overall, the group was predominantly male (93.9%) and obese (median BMI 32.2 [IQR 8.1]), with a low prevalence of current tobacco smoking (12.6%). There were 262 (16.6%) who carried a diagnosis of concomitant lung disease at the time of the referral. By diagnosis, these were: COPD (*N* = 94; 6.0%); asthma (*N* = 181; 11.5%); and interstitial lung disease (*N* = 8; 0.5%). Those with moderate to severe OSA compared to those with mild or no disease on average were 6-years older, had a higher BMI, were more likely to be male, more likely to be married or partnered, and were less likely currently employed (all *p* < 0.05, Table 1). There were no statistically significant differences by OSA status for race-ethnicity or educational attainment. In terms of concomitant lung disease, those with moderate to severe OSA were statistically more likely to have concomitant ILD but not COPD or asthma.

**Table 1:** Subject characteristics by obstructive sleep apnea status^a^

	All subjects	None to mild OSA^b^	Moderate to severe OSA	
Subject characteristics	*N* = 1578	*N* = 755	*N* = 823	*p*-value
Age, years	61.1 (16.8)	58 (22.2)	63.3 (13.5)	<0.001
Male, n (%)	1482 (93.9%)	686 (91.9%)	796 (96.7%)	<0.001
Ethnicity				
White	996 (63.1%)	480 (63.6%)	516 (62.7%)	0.72
Non-white	582 (36.9%)	275 (36.4%)	307 (37.3%)	
<High School Education	325 (20.6%)	154 (20.4%)	171 (20.8%)	0.85
Married/partnered	1039 (65.8%)	469 (62.1%)	570 (69.3%)	<0.01
Currently Employed	558 (35.4%)	289 (38.3%)	269 (32.7%)	<0.05
BMI, kg/m^3^	32.2 (8.1)	30.6 (6.3)	34.3 (8.8)	<0.001
Smoking status				0.03^c^
Never	553 (35.1%)	280 (37.1%)	273 (33.2%)	
Former	826 (52.3%)	369 (48.9%)	457 (55.5%)	
Current	199 (12.6%)	106 (14.0%)	93 (11.3)	
Any Lung Disease	262 (16.6%)	129 (17.1%)	133 (16.2%)	0.62
COPD	94 (6.0%)	50 (6.6%)	44 (5.3%)	0.29
Asthma	181 (11.5%)	90 (11.9%)	91 (11.1%)	0.59
ILD	8 (0.5%)	0 (0%)	8 (1.0%)	<0.01^d^

^a^ *Abbreviations*: *BMI* Body Mass Index, *COPD* chronic obstructive pulmonary disease, *ILD* interstitial lung disease, *OSA* obstructive sleep apnea

^b^None or mild OSA defined as an apnea-hypopnea index <15 events per hour. Moderate and severe obstructive sleep apnea defined as an apnea-hypopnea index ≥15 events per hour

^c^ *P*-value for 2*3 χ^2^ test. *P*-value for 2*2 test comparing ever with never smokers is 0.10

^d^ *P*-value for Fisher exact 2-sided test

The median overall ESS score was 11 [IQR 9], with 838 (53.1%) falling above the 10-point score cut off for increased daytime sleepiness. The overall FSS score was 42 [IQR 23], with 968 (61.3%) falling above the 36-point score cut off for increased fatigue. The ESS and FSS scores were moderately intercorrelated (*r* = 0.40; *p* < 0.01). The correlations between ESS and FSS when stratified by OSA status were: *r* = 0.37 among those with mild to no OSA and *r* = 0.46 among those with moderate or severe OSA.

Overall, the study population reported low general HRQL, with a median SF-12v2 PCS of 40.3 [IQR 21.9]. There was no statistical difference in SF-12v2 PCS by OSA status (none or mild vs. moderate to severe): 40.6 [IQR 22.3] vs. 40.2 [IQR 21.6], respectively. For the entire group, respiratory-specific HRQL measured by the AQ-20R, yielded a median value of 4 (IQR 1–8; absolute range 0–20). Those with moderate to severe OSA compared to those with milder or no disease manifested slightly higher respiratory-specific HRQL scores consistent with poorer status, but this difference was not statistically significant (Wilcoxon rank-sum test’s *p* = 0.11).

We tested sleepiness (ESS), fatigue (FSS), and the degree of OSA (AHI) as predictors of physical and respiratory specific HRQL, taking into account other covariates (Table 2). Both ESS and FSS were significantly associated with poorer SF-12v2 PCS (*p* < 0.01), although the point estimate of effect was greater for the latter (−3.3 and −7.6 point lower PCS per standardized units of ESS and FSS, respectively). A similar pattern was evident for respiratory-specific HRQL measured by the AQ-20R. In contrast, AHI was not associated with SF-12v2 PCS or AQ-20R.

**Table 2:** Sleepiness, fatigue, and sleep apnea in relation to heath-related quality of life

	Linear regression modeling									
	Model 1^a^					Model 2^b^				
Risk factor	Beta	95% CI	*p*-value	Partial R^2^	Total R^2^	Beta	95% CI	*p*-value	Partial R^2^	Total R^2^
Short Form (SF)-12 Physical Component Scale										
ESS	−3.33	−3.99;−2.68	<0.01	0.06	0.09	−0.50	−1.11;0.12	0.11	<0.01	0.33
FSS	−7.56	−8.13;−7.00	<0.01	0.31	0.33	−7.37	−7.98;−6.76	<0.01	0.26	
AHI	−0.11	−0.86;0.64	0.77	<0.01	0.04	0.08	−0.55;0.71	0.81	<0.01	
AQ-20R Respiratory-Specific Health-Related Quality of Life										
ESS	1.62	1.39;1.86	<0.01	0.10	0.12	0.78	0.54;1.02	<0.01	0.03	0.27
FSS	2.48	2.26;2.70	<0.01	0.24	0.25	2.18	1.94;2.41	<0.01	0.17	
AHI	0.17	−0.11;−0.45	0.23	<0.01	0.02	0.04	−0.20;0.29	0.73	<0.01	

All regression beta values are scaled standardized units of the independent predictors. Higher SF-12 equates with better Health-Related Quality of Life; higher AQ-20R with poorer health-related quality of life

*Abbreviations*: *AHI* apnea-hypopnea index, *BMI* Body Mass Index, *CI* confidence intervals, *ESS* Epworth Sleepiness Scale, *FSS* Fatigue Severity Scale

^a^Model 1 also includes age, sex, marital status and BMI

^b^Model 2 includes ESS, FSS, AHI altogether in the same multivariate model, along with age, sex, marital status and BMI

**Fig. 1:**
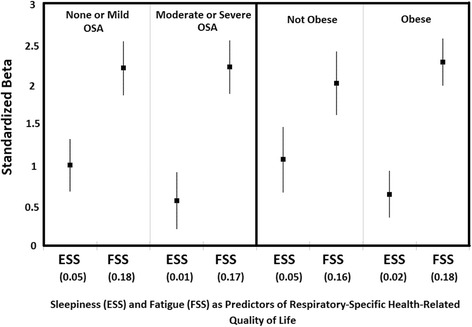
Multiple Regression Modelling of Sleepiness and Fatigue in Relation to Respiratory-Specific Health-Related Quality of Life (AQ-20R) Stratified by the Presence or Absence of Moderate to Severe Sleep Apnea or Obesity. Regression beta values and associated 95% confidence intervals are shown. Predictors are shown with their partial R2 in brackets. Abbreviations: ESS (Epworth Sleepiness Scale), FSS (Fatigue Severity Scale), OSA (obstructive sleep apnea). None or mild obstructive sleep apnea defined as an apnea-hypopnea index <15 events per hour. Moderate or severe obstructive sleep apnea defined as an apnea-hypopnea index ≥15 events per hour. Obesity defined as a body mass index ≥ 30 kg/m2. All multivariate models include age, sex, marital status, and body mass index, as well as the ESS and FSS scales. All regression beta values are scaled in standardized units of the independent predictors. All beta coefficients *p* < 0.001. Overall model R^2^ values are: none-mild OSA, 0.28; moderate to severe OSA, 0.26; not obese, 0.27; obese, 0.26

In analyses stratified by the presence or absence of OSA (Fig. 1), increased fatigue was associated with poorer respiratory HRQL in both the none-to-mild and the moderate-to-severe OSA groups. In comparison, daytime sleepiness was not associated with poorer respiratory-specific HRQL in those with none-to-mild OSA and demonstrated an attenuated effect (standardized beta 0.6 for ESS compared to 2.2 for FSS) in those with moderate-to-severe OSA. Also as shown in Fig. 1, the pattern was similar when stratified by the presence or absence of obesity.

We further explored the associations of ESS and FSS with respiratory-specific HRQL, taking into account generic HRQL (SF-12v2 PCS), OSA severity, comorbid chronic lung conditions, and the covariates included in the previous multivariate analyses (Table 3). Both ESS and FSS remained significantly associated with AQ-20R, the FSS manifesting a somewhat greater effect. SF-12v2PCS and comorbid lung conditions were associated with poorer respiratory specific quality of life measured by the AQ-20R, whereas presence or severity of OSA was not. Excluding 1275 subjects with portable sleep study testing (remaining *n* = 303) had no substantial effect on these multivariate analyses except that the associations with COPD (*p* = 0.06) and ILD (*p* = 0.94) were no longer statistically significant.

**Table 3:** Obstructive sleep apnea, sleepiness, and fatigue as risk factors for poorer respiratory-specific health-related quality of life

Risk factor	OR	95% CI	*p*-value
OSA [Referent = None]			
Mild	1.28	0.81; 2.01	0.29
Moderate/severe	1.19	0.76; 1.86	0.45
ESS	1.37	1.17; 1.60	<0.01
FSS	2.38	1.90; 2.99	<0.01
SF-12 PCS	1.79	1.47; 2.17	<0.01
Asthma	3.78	2.56; 5.59	<0.01
COPD	2.59	1.51; 4.41	<0.01
ILD	26.53	2.72; 258.46	<0.01

Poor respiratory-specific QOL defined as the 20% highest AQ-20R scores, 11 through 20

Mild OSA defined as apnea-hypopnea index greater than 5 but lower to 15. Moderate or severe obstructive sleep apnea defined as apnea-hypopnea index equal as or higher than 15

ESS, FSS, PCS of the Short Form-12 are scaled per standard deviation units for comparability among scales. The PCS of the Short Form-12 is reversed scored for this analysis to indicate risk with poorer generic quality of life

Age, sex, marital status and BMI were included in the model (other than younger age (OR 0.99, *p* = 0.03), none were statistically significantly associated with poorer QOL)

*Abbreviations*: *BMI* Body Mass Index, *ESS* Epworth Sleepiness Scale, *FSS* Fatigue Severity Scale, *OR* Odds Ratio, *OSA* Obstructive Sleep Apnea, *PCS* Physical Component Scale of the Short Form-12, *QOL* Quality of Life

## Discussion

The assessment of HRQL, a patient-centered outcome encompassing health, functional, and social factors, is critical to inform individual patient management decisions and, in the aggregate, health care policy. Asthma, COPD, and interstitial lung disease all are chronic respiratory conditions associated with poorer HRQL [24]. Given the potential combined effects from comorbid OSA and chronic respiratory disorders such as these, evaluating generic and respiratory HRQL in populations with multiple morbidities is important.

Our analysis, carried out in a relatively large sample of military veterans undergoing sleep evaluations for suspected OSA, yielded two key findings. First, sleepiness and fatigue were important determinants of respiratory-specific HRQL, independent of OSA, respiratory comorbidity, or even generic physical HRQL. Second, OSA severity *per se* was not associated with a significant decrement in either generic physical HRQL or respiratory-specific HRQL. Although the lack of a strong association of OSA with physical HRQL has been reported [11, 30], respiratory-specific HRQL previously had not been studied in parallel. Thus taking these two findings together, among those with symptoms or clinical findings suggesting OSA, regardless of the ultimate diagnosis, fatigue and sleepiness negatively impact the key patient-centered outcome of respiratory-specific HRQL.

Although untreated OSA may be associated with poorer sleep-specific quality of life, both general population and sleep clinic-based studies have reported inconsistent associations between OSA severity and generic physical HRQL [5–7, 9–11, 30, 31]. Additionally, studies evaluating symptomatic patients referred for OSA assessment report similar findings to our investigation. Among 109 patients with suspected OSA referred for PSG testing (mean age 53 years, 77% male, BMI 31, AHI 33.6), ESS was negatively associated with physical HRQL measured by the SF-12 PCS (*p* < 0.001), whereas AHI was not associated with that outcome [6]. In another series of 135 consecutive patients with suspected OSA, ESS was associated with generic physical HRQL, but not the AHI or EEG arousal index [10]. Other referral series also fail to show a relationship between AHI and physical HRQL [5, 7, 11]. Given these findings and our own, there appears to be no clear association between OSA severity (as measured by AHI) and generic physical HRQL, even though subjective sleepiness (measured by the ESS) indeed is correlated consistently with HRQL. This interpretation is consistent with another study in which an association of general HRQL with ESS was observed, but not with OSA [10].

Our study goes further still by also taking into account other respiratory co-morbidities as well as other symptoms such as fatigue. Even when patients with active medical and psychiatric disorders are excluded, ESS but not AHI, respiratory disturbance index or oxygen desaturation index is still negatively associated with physical HRQL measured by the SF-12 PCS [30].

Similar to our results, studies in patients with chronic respiratory disorders such as COPD report no correlation between AHI and respiratory-specific HRQL measured by the St. George Respiratory Questionnaire (SGRQ) [12]. It is well established that both generic and respiratory-specific HRQL are poor among patients with asthma and COPD [15, 16, 19, 32]. However, the interrelationships among OSA, respiratory comorbidity, sleepiness, and respiratory-specific HRQL are less clear-cut. This interrelationship is likely to be complex given that asthma and COPD are associated with poor sleep quality that may be manifested as daytime sleepiness, fatigue, and poorer general HRQL, independent of OSA [2, 12, 16, 33, 34]. Our findings support those links, but adds to picture by taking into account both OSA and other chronic respiratory comorbid conditions, yet still observing a potent negative relationship between increased daytime sleepiness and fatigue and respiratory-specific HRQL. Standardizing the scoring metric, we observed a greater negative impact of fatigue relative compared to daytime sleepiness on generic and respiratory-specific HRQL. Studies suggest subjective fatigue and sleepiness are indeed distinct entities [35], albeit not wholly independent consistent with our finding that FSS and ESS scores were moderately intercorrelated (*r* = 0.38). Some studies have reported that AHI is poorly correlated with fatigue [36, 37], whereas other chronic respiratory disorders are linked to fatigue [38, 39]. Thus, our analysis supports a growing consensus that fatigue negatively impacts generic and respiratory-specific HRQL while specifically showing that this relationship transcends OSA and even other comorbid respiratory disease.

Our study supports the utility of the AQ20-R in measuring respiratory-specific HRQL in OSA. The AQ20 was developed as a simple respiratory HRQL instrument that correlates well with other respiratory (SGRQ) and general (SF-12 and SF-36) HRQL measures in populations with various types of respiratory disorders [24, 25]. Additionally, the AQ20-R is an independent predictor of healthcare utilization outcomes in those with chronic respiratory disease [24]. Our study is novel in assessing the impact of subjective fatigue and sleepiness on respiratory HRQL measured by the AQ20-R. Consistent with our findings, fatigue (measured by the Multidimensional Fatigue Inventory) strongly correlated with respiratory-specific HRQL measured by the SGRQ (*r* = 0.75, *p* < 0.01) [20]. In patients with chronic respiratory disorders, there appears to be a complex inter-relationship among fatigue, sleep quality, anxiety, depressive symptoms, and awake-dyspnea [40]. Interestingly, the AQ20 appears moderately correlated with subjective sleep disturbance in asthmatics (*r* = 0.51, *p* ≤ 0.001) [34], while both non-respiratory medical and psychiatric comorbidities are independent modulators of HRQL measured by the AQ20-R [41].

Taken together, the clinical implications of our observations are that the evaluation and treatment of respiratory complaints among those with suspected OSA is important, particularly in those reporting fatigue. The AQ20-R assesses respiratory-specific HRQL. Taking this or comparable disease-specific patient-centered measures of health into account in models of health care delivery is likely to be important for clinical management and improved patient satisfaction among those with suspected OSA, whether or not they ultimately prove to have that condition.

Our study has potentially important limitations. These limitations could argue against the generalizability of our findings, the consistency of our observations with those of other relevant studies, our emphasis on respiratory–specific or even general physical status as opposed to mental HRQL, or the validity of our definition of disease. We evaluated generally older obese male military veterans referred for suspected OSA, which should temper any general population inferences. However, studies in populations consisting of older, primarily male non-veterans with suspected OSA report similar results to ours [6, 10, 30]. Second, the applicability of general physical HRQL measures such as the SF-12 in OSA populations has been questioned, particularly when comorbid medical and psychiatric conditions are not controlled for [42], and because sleep and OSA-specific HRQL batteries correlate only modestly with generic HRQL [43–45]. Additionally, generic mental health rather than physical HRQL may correlate with AHI [46, 47]. We limited our analysis of the SF-12 to the PCS and did not investigate associations with the SF-12 Mental Component Scale (that measures psychological HRQL), nor did we study psychological comorbidity. Third, in our bivariate analysis, pooling mild OSA together with no OSA to compare this categorization against moderate-severe OSA could lead to disease misclassification. The Sleep Heart Health Study, however, reported no clinically relevant differences in SF-36 and ESS scores between those with mild vs. no OSA [9, 48] and the recent Apnea Positive Pressure Long-term Efficacy Study similarly reported no significant differences in ESS and Sleep Apnea Quality of Life Index between those with mild vs. no OSA [49]. Finally, given limited spirometric data, we could not analyze the association of respiratory impairment (as opposed to the presence or absence of a diagnosed respiratory condition) with HRQL. Considering these potential limitations as whole, they are modest at most and are unlikely to explain the associations that we observed, argue strongly for an alternative explanation of the findings of this analysis, or seriously undermine the credibility of our findings.

## Conclusions

In summary, daytime sleepiness and fatigue contribute to poorer respiratory-specific HRQL, taking into account the effects of OSA, respiratory comorbidity, and generic HRQL. The clinical implications of this analysis are that the evaluation and management of non-OSA causes of fatigue, sleepiness, and daytime breathing problems are relevant to patients with complaints suggesting a diagnosis of OSA. This is the case whether or not such patients ultimately prove to have OSA or, if they do, the degree of its severity. Furthermore, we believe the AQ20-R provides additional utility in HRQL assessments as well as being a simple patient-centered measure of breathing problems in those suspected of having OSA. Future longitudinal assessments should evaluate whether respiratory-specific HRQL, with or without concomitant lung disease, predicts subsequent health outcomes in OSA populations and the extent to which fatigue or sleepiness account for such relationships.
